# The Place of Dipeptidyl Peptidase-4 Inhibitors in Type 2 Diabetes Therapeutics: A “Me Too” or “the Special One” Antidiabetic Class?

**DOI:** 10.1155/2015/806979

**Published:** 2015-05-17

**Authors:** Ricardo Godinho, Cristina Mega, Edite Teixeira-de-Lemos, Eugénia Carvalho, Frederico Teixeira, Rosa Fernandes, Flávio Reis

**Affiliations:** ^1^Laboratory of Pharmacology and Experimental Therapeutics, Institute for Biomedical Imaging and Life Sciences (IBILI), Faculty of Medicine, Coimbra University, 3000-548 Coimbra, Portugal; ^2^ESAV, Polytechnic Institute of Viseu, 3504-510 Viseu, Portugal; ^3^Center for Neuroscience and Cell Biology-Institute for Biomedical Imaging and Life Sciences (CNC.IBILI) Research Unit, University of Coimbra, 3000-548 Coimbra, Portugal; ^4^The Portuguese Diabetes Association (APDP), 1250-189 Lisbon, Portugal

## Abstract

Incretin-based therapies, the most recent therapeutic options for type 2 diabetes mellitus (T2DM) management, can modify various elements of the disease, including hypersecretion of glucagon, abnormal gastric emptying, postprandial hyperglycaemia, and, possibly, pancreatic *β* cell dysfunction. Dipeptidyl peptidase-4 (DPP-4) inhibitors (gliptins) increase glucagon-like peptide-1 (GLP-1) availability and correct the “incretin defect” seen in T2DM patients. Clinical studies have shown good glycaemic control with minimal risk of hypoglycaemia or any other adverse effects, despite the reports of pancreatitis, whose association remains to be proved. Recent studies have been focusing on the putative ability of DPP-4 inhibitors to preserve pancreas function, in particular due to the inhibition of apoptotic pathways and stimulation of *β* cell proliferation. In addition, other cytoprotective effects on other organs/tissues that are involved in serious T2DM complications, including the heart, kidney, and retina, have been increasingly reported. This review outlines the therapeutic potential of DPP-4 inhibitors for the treatment of T2DM, focusing on their main features, clinical applications, and risks, and discusses the major challenges for the future, in particular the possibility of becoming the preferred therapy for T2DM due to their ability to modify the natural history of the disease and ameliorate nephropathy, retinopathy, and cardiovascular complications.

## 1. Incretins in Healthy and Disease: Overview

### 1.1. The Incretin Effect

The gastrointestinal (GI) tract contains a multitude of regulatory peptides that transmit information not only to the intestine and associated organs, but to other systems, such as the central nervous system (CNS) and the cardiovascular system. At the beginning of the 20th century, experiments were carried out with mucosa extracts of the small intestine for treatment of diabetes mellitus, based on the idea that gastrointestinal hormones stimulated pancreatic endocrine function [1]. Historically, Bayliss and Starling in 1905 examined the effects of crude intestinal extracts on exocrine pancreatic secretions and reported the existence of a “secretin,” the first regulatory peptide to be identified, thus introducing the concept of hormones and describing their way of action [2]. In 1906, Moore et al. discovered a chemical stimulant produced by the pancreas and, in 1930, La Barre studied the effects of intravenous administration of unclean “secretin” on blood glucose levels. The incretins identified in the 1930s were associated with intestinal synthesis of hormones similar to insulin and were, thus, responsible for the introduction of this term, which was originated from the junction of fragments of the words “IN”testin, se“CRET”ion, and “IN”sulin. Basically, an incretin describes a factor that reduces blood glucose levels without affecting exocrine pancreatic secretion. However, it would take more than 30 years before they showed perceptive implications on the regulation of blood glucose. Mcintyre et al. [3] were the first to demonstrate the “incretin effect” in 1964, by observing that oral administration of glucose caused a greater increase in insulin secretion than the same amount of glucose administered intravenously, despite the higher blood glucose levels registered by the intravenous route. Oral glucose administration resulted in increased insulin secretion, confirming the existence of a link between the intestine and the endocrine pancreas, leading to the assumption that gastrointestinal hormones could have an additional action on insulin secretion. Therefore, for a given hormone to be included in a group of “incretins,” it must meet two essential criteria [4]: be released in response to oral glucose intake and be able to achieve physiological concentrations resulting in insulin release.

The revival of the term incretin was mostly due to Creutzfeldt [2, 5], who emphasized the relationship “glucose-insulin-intestine” in association with the incretin effect, a feature that is of profound importance for its clinical application. In 1986, Nauck et al. studied the incretin effect (insulin response after oral versus intravenous administration of either 25, 50, or 100 g of glucose) by measuring the concentrations of C-peptide, a marker of endogenous insulin secretion [6]. These investigators found that the level of incretin secretion was dependent on the amount of ingested glucose and that incretins were responsible for approximately 75% of the insulin response after the ingestion of 50 g of glucose.

The study of incretin hormones was pursued by a number of researchers, but identification of the first incretin came from an unexpected source. Brown of the University of British Columbia, Vancouver, Canada, tried to isolate a hormone involved in the regulation of gastric acid secretion from pig intestinal extracts: enterogastrone. In collaboration with other researchers, he identified and isolated a hormone composed of 42 amino acids, to which he gave the name of “gastric inhibitory polypeptide” (GIP) [6], now also known as “glucose-dependent insulinotropic polypeptide,” since it was shown to be able to stimulate insulin secretion in a glucose-dependent manner; it is an incretin [7]. Later, a second incretin was isolated due to genetic studies on proglucagon coding sequences, and it was described as a “glucagon-related molecule” [8]. This molecule was named “glucagon-like peptide-1” (GLP-1) and as it met the criteria, it was classified as an incretin.

Both incretins are secreted in the intestinal mucosa; GIP is secreted from the K-cells (enterochromaffin cells) located mainly in the stomach, duodenal mucosa, and the proximal jejunum, whereas L-cells produce GLP-1 and are located more distally in the ileum and colon [9]. Within minutes of nutrient ingestion, both incretins (GIP and GLP-1) are released into the bloodstream and stimulate insulin secretion [10]. The metabolic, hormonal, and neuronal influences on the endocrine pancreas are collectively referred to as the “enteroinsular axis” [11]. Postprandial insulin secretion is directly stimulated by substrates of nerve stimulation, through enteropancreatic nerve activation by chyme and intestinal distension, and by a strong endocrine stimulus mediated by incretin hormones.

Besides GIP and GLP-1, several other gastrointestinal hormones are released from endocrine cells and neurons in the digestive tract, which makes the gut the largest hormone producing organ in the body. The physiological functions of these peptides have been revealed during the last years, namely, those concerning the regulation of glucose homeostasis and their putative use as therapeutic target for obesity, and diabetes is an emerging and evolving challenge, as previously reviewed [12–14] and briefly revisited in this section. Ghrelin is secreted from the stomach in the fasting state and is an appetite-stimulating GI hormone, also known as “the hunger hormone.” Cholecystokinin (CCK) is mainly produced in the L-cells of the duodenum and small intestine in response to a meal, thus stimulating pancreatic hormone and bile secretion and inhibiting gastric emptying; CCK was the first GI hormone found to act as a hunger suppressant. The classical action of gastrin is the control of gallbladder contraction, satiety, and pancreatic and gastric acid secretion, but current knowledge points to participation in the control of glucose homeostasis, namely, by stimulation of glucagon release from human islets in vitro. Peptide YY (PYY) is produced in the GI L-cells, mainly in the colon and rectum, and has been viewed as a meal “termination” signal and shows “satiety peptide” properties and its levels are low after overnight fasting and elevated after meal. PYY belongs to the “PP fold” family of peptides which includes pancreatic polypeptide (PP) and neuropeptide Y (NPY). PP is secreted in the islets of Langerhans and, in smaller amounts, by colon and rectum cells and has been associated with reduction of gastric emptying. Its fasting levels are low, but their postprandial levels increase and are correlated with meal calorie content. Oxyntomodulin (OXM) is secreted by the L-cells, in parallel with GLP-1 production, and shows an incretin effect, reducing the appetite and the amount of ingested food, an effect that seems to be partly due to the inhibition of ghrelin secretion. Amylin, also known as islet amyloid polypeptide (IAPP), is secreted together with insulin in pancreatic *β* cells and has been suggested to play a role in glucose homeostasis by suppressing the release of glucagon from pancreatic *α* cells, thus preventing the release of glucose from the liver, decreasing the gastric emptying, and stimulating the satiety center in the brain [15]. Although several GI hormones have been demonstrating impact on glucose metabolism, incretins hormones, mainly GLP-1, have been used as therapeutic target for diabetes treatment and will be the focus of this paper.

Approximately 30 to 60% of C-peptide and 80 to 90% of the insulin response after an oral glucose load are regulated by incretin hormones, depending on the amount of glucose [6]. Incretin action on pancreatic *β* cells involves a series of events that potentiate the action of glucose, an important feature that is protective against the development of hypoglycaemia. One of the characteristics of these incretins, which makes them attractive as potential therapeutic agents, is that the associated insulin secretion ceases when euglycemia is achieved, thus minimizing the risk of hypoglycaemia [16]. Both GIP and GLP-1 exert their effects by binding to their specific receptors, the GIP receptor (GIPR) and the GLP-1 receptor (GLP-1R), stimulating a cascade of events that culminate in stimulation of glucose-dependent insulin secretion in pancreatic *β* cells. GIP and GLP-1 are rapidly metabolized (*t*_1/2_ ≈ 2 minutes) [17] by the ubiquitous enzyme dipeptidyl peptidase-4 (DPP-4) to inactive metabolites and then eliminated by the urine [18]. GIP and GLP-1 share common properties as incretins, but they also possess different biological characteristics. GLP-1 acts in a positive way on the *β* and *δ* cells, whereas GIP acts preferentially on the *α* and *β* cells [18]. The effects of GLP-1 on pancreatic islet cells include increased insulin secretion by *β* cells in a glucose-dependent manner, increased secretion of somatostatin by *δ* cells, and reduced secretion of glucagon by the *α* cells. These measures contribute to a decrease in the hepatic glucose output. In addition to their insulinotropic effects, GIP and GLP-1 play critical roles in various biological processes in different tissues and organs that express GIPR and GLP-1R, including the pancreas, adipose tissues, bone, peripheral and central nervous systems (CNS), heart, kidney, liver, and GI tract [19, 20].

**Figure 1:**
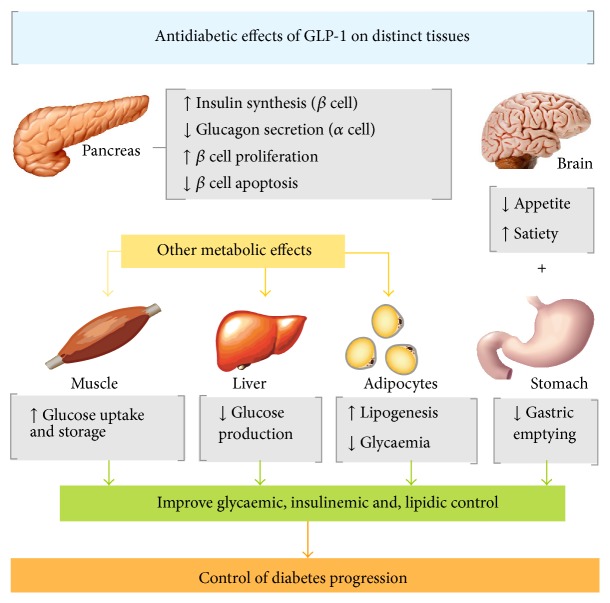
Antidiabetic insulin-dependent and insulin-independent effects of GLP-1 on metabolic tissues, which are potentiated by inhibition of dipeptidyl peptidase-4, thus improving the glycaemic, insulinemic, and lipidic profile and the progression of the disease.

Here, we briefly revise the similarities and differences concerning the GLP-1 and GIP insulinotropic actions on pancreatic *β* cells and their noninsulinotropic effects on pancreas and on extrapancreatic tissues, which have been revealed during the last years [21, 22].  Figure 1 schematically presents the major biological actions of GLP-1 on pancreas and on tissue involved in their metabolic antidiabetic effects.

### 1.2. Biological Effects of GLP-1 and GIP on Distinct Tissues

#### 1.2.1. Effects on Pancreas

*(a) Insulinotropic Actions of GLP-1 and GIP.* One of the most important properties of GIP and GLP-1 is their ability to promote insulin secretion, maintaining glucose homeostasis without inducing hypoglycaemia. Both GIP and GLP-1 are key mediators/regulators of pancreatic function and pancreatic *β* cell mass. Human beings spend most of their time in a postprandial state, and therefore it is important to emphasize that these peptides are almost undetectable during fasting and exist at high concentrations in the postprandial state. GLP-1 and GIP promote glucose-dependent insulin secretion and insulin biosynthesis, acting to regulate postprandial glucose disposal [21]. Binding of GIP and GLP-1 to their specific receptors (GIPR and GLP-1R, resp.) leads to the activation of adenylate cyclase and subsequent elevation of intracellular cyclic adenosine monophosphate (cAMP) levels, which then activates a signalling cascade that causes the increment of intracellular Ca2+ concentrations triggering the fusion of insulin-containing granules with the plasma membrane and insulin secretion from the *β* cells. Increased Ca2+ levels also promote transcription of the proinsulin gene, thereby increasing the insulin content of the *β* cell [23]. Further studies should clarify the differences in signaling events downstream of GIPR and GLP-1R.

Another important aspect of the insulinotropic effects of GIP and GLP-1 is their synergy with the sulfonylurea drugs, which is clinically relevant due to the risk of hypoglycemia when used in combined therapies [24, 25].

*(b) Noninsulinotropic Actions of GLP-1 and GIP*

*(i) On Pancreatic β Cells.* Although the major role of GIP and GLP-1 has generally been thought to stimulate insulin secretion by pancreatic *β* cells, it is now known that GIP and GLP-1 exert noninsulinotropic actions, such as controlling pancreatic *β* cell proliferation and survival. Both hormones seem to be associated with antiapoptotic and proproliferative effects on pancreatic *β* cell, but the signaling cascades involved display some differences that have been previously revealed with more details [21–23, 26–28]. A critical difference in the antiapoptotic function of GLP-1 is the requirement for PI3K, which is not required for the antiapoptotic action of GIP, whose physiological impact remains to be fully clarified [29]. Another important aspect of GIP and GLP-1 action on *β* cells is the stimulation of the proliferation of *β* cells and/or progenitor cells [30–32]. Stimulation of *β* cell proliferation and inhibition of apoptosis promote cell expansion, which was observed in diabetic mice and in *β* cell cultures [33]. GLP-1 has a trophic action on *β* cells in terms of amplification of insulin synthesis and in respect of *β* cell hypertrophy. GLP-1 increases *β* cell mass by stimulating cell proliferation, inducing pancreatic islet neogenesis, and inhibiting cellular apoptosis [20]. GLP-1 also promotes cell differentiation, from exocrine ductal cells or immature islet stem cells, towards a greater degree of differentiation [34]. An increase in the number and mass of *β* cells has been demonstrated by direct action of GIP [33, 34]. Further elucidation on the precise molecular mechanisms underlying the effects of GIP and GLP-1 on *β* cell could reveal potential therapeutic targets to increase *β* cell mass by inhibiting apoptosis and/or stimulating proliferation.

*(ii) Effects on Glucagon and Somatostatin Secretion from Pancreatic α and γ Cells.* The effects of GLP-1 and GIP on glucagon secretion from pancreatic *α* cells are opposing. In contrast to GIP, GLP-1 inhibits the release of both somatostatin and glucagon. Inhibition of somatostatin is mediated through direct effects on the pancreatic *γ* cell [35]. GLP-1 suppresses glucagon secretion when plasma glucose levels are above fasting level [36], which is clinically important because GLP-1 loses its inhibitory effect on glucagon secretion at hypoglycemic levels and does not attenuate the counterregulatory responses to hypoglycemia. Furthermore, it has been recently reported that insulin stimulation and glucagon inhibition contribute equally to the effect of GLP-1 on glucose turnover in T2DM patients [37]. Although there are no GLP-1 receptors on *α* cells, insulin released by *β* cells, in response to GLP-1, turns out to have an inhibitory action on the physiological secretion of glucagon. Therefore, there is an improvement, at least partial, in the insulin/glucagon ratio, which improves glucose uptake by the liver and peripheral tissues, such as skeletal muscle. Despite its clinical importance, the mechanism underlying the suppression of glucagon secretion by GLP-1 remains to be clarified. In contrast with GLP-1 effect, infusion of GIP was shown to counteract suppression of glucagon secretion by glucose, which was observed in rats and further confirmed in healthy humans during euglycemic, but not during hyperglycemic, clamp studies, as well as in T2DM patients [38–41]. Although its physiological importance remains unknown, enhancement of glucagon secretion by GIP hinders clinical usage of GIP as T2DM treatment.

#### 1.2.2. Effects on Adipose Tissue

GIP has been proposed to have a physiological role in nutrient uptake into adipose tissues, thereby linking overnutrition to obesity. GIP levels are high in obese T2DM patients and fats strongly enhance GIP secretion [42–44]. The role played by GIP in adipose tissue has been revealed during the last years. The first clue came from the evidence of fatty acid incorporation into rat epididymal fat pads induced by GIP in the presence of insulin [45]. These initial evidences were supported by GIPR expressed in adipose tissues [46] and then reinforced by studies of genetic ablation of GIPR, which clarified some of the critical roles played by GIP in fat accumulation [47]. In fact, GIPR-deficient mice fed high-fat diets showed higher energy expenditure indicating preferential use of fat as energy substrate, together with increased adiponectin secretion which promotes fat oxidation in muscle and increases the respiratory quotient [48, 49]. In obese ob/ob mice, in which a defect in the leptin gene results in hyperphagia and subsequent obesity [50], genetic ablation of GIPR improved not only obesity by increasing energy expenditure [47, 51], but also insulin insensitivity and glucose tolerance without seriously affecting insulin secretion [52]. These findings were confirmed when a GIPR antagonist was used in high-fat fed mice and in obese ob/ob mice treated [53–56].

Although GIP was shown to increase the activity of lipoprotein lipase (LPL), which hydrolyzes lipoprotein-associated triglycerides to produce free fatty acids available for local uptake [47], the molecular mechanism by which GIP acts on adipocytes is largely unknown. Further investigation might shed light on the molecular mechanisms underlying GIP action in fat accumulation and might open up a possibility of GIP-based antidiabetic therapy that does not promote obesity. Importantly, GLP-1 does not show any role in fat accumulation. While GLP-1R is expressed in adipocytes [57], activation of GLP-1R affects none of the aforementioned signaling molecules and does not increase LPL activity in adipocytes [58, 59]. However, the insulin secretion evoked by GLP-1 and the consequent suppression of the release of fatty acids is probably the most dominant effect observed on adipose tissue, with a simultaneous stimulation of glycogen synthesis.

#### 1.2.3. Effects on Stomach-Brain Regulation of Appetite and Satiety

GIP and GLP-1 receptors are present in the stomach. Although GLP-1 inhibits gastric emptying [60, 61], GIP has been shown to have little effect on gastric emptying in humans and mice [41, 62]. GLP-1 slows gastric emptying and inhibits pentagastrin and meal-stimulated gastric acid secretion [63, 64]. Stimulation of GLP-1 receptors in the pyloric sphincter causes a deceleration of gastric emptying and reduces postprandial blood glucose [65]. Delaying gastric emptying and maintaining subsequent distension of the stomach affects peripheral satiety signals. Turton et al. (1996) [66] examined the satiety effect of GLP-1 on the central CNS in fasted rats injected intracerebrally, in the ventricular area, with GLP-1 versus saline (control group), and measured food consumption at regular intervals. Food intake decreased progressively with the increase of the concentration of injected GLP-1, whose receptors were detected within different areas of the brain, including densely innervate hypothalamic regions, such as the paraventricular, dorsomedial, and arcuate nuclei [34, 67]. In the presence of food, GLP-1 may mediate gut-brain signalling from the gastrointestinal tract to GLP-1 receptors in the hypothalamus and brainstem. This constitutes a feeding control via neural and endocrine mechanisms [68, 69].

Several lines of evidence imply that not only GLP-1, but also GIP, controls food intake and satiety. GIPR deficiency seems to prevent ovariectomy-induced obesity, which might be linked to the reduced expression of NPY in the hypothalamus and subsequent reduction of food intake [70]. In fact, it was previously shown that cerebral infusion of NPY stimulates neuronal secretion of GIP, suggesting that GIP acts as a negative regulator of NPY, thus controlling food intake [71]. Thus, the antiobesity function of GIP might result not only from the aforementioned effects on the adipose tissues but also from a direct effect on the brain. Similar evidences have been obtained for GLP-1, such as the inhibition of food intake [66, 72] by intracerebroventricular and peripheral infusion of GLP-1R agonists, and further confirmed using the GLP-1 and GLP-1R antagonist exendin-(9–39) [73, 74].

#### 1.2.4. Effects of GLP-1 and GIP on Other Organs

In addition to the pancreas, adipose tissue, stomach, and brain, receptors for GIP and GLP-1 are expressed in a wide variety of organs, including the bones, heart, and kidneys, where incretins seem to play important effects. Regulation of bone metabolism is another important physiological function of incretins. GIPR are expressed in bones and GIP directly affects bone metabolism, having a role in bone formation, as suggested by the reduction of bone formation parameters and high turnover osteoporosis in GIPR-deficient mice [75, 76]. Unlike GIP, GLP-1 has no direct effect on osteoblasts and osteoclasts, and GLP-1 inhibits bone resorption indirectly through upregulation of calcitonin [77, 78]. Although exendin-4 has been shown to promote bone formation in rats [79], whether GLP-1-based therapies show any effects on bone metabolism in human remains to be addressed in the future.

Recent studies have shown that incretins can regulate other vital functions, such as body temperature, blood pressure, heart rate, and fluid balance. The main cardiovascular and renal effects described will be reviewed in further sections of this paper. In addition, there is increasing interest in the potential role of incretin hormones in neurodegenerative disorders, including Alzheimer's, Parkinson's, and Huntington's diseases [80–83].

### 1.3. The “Incretin Defect” in T2DM

GLP-1 is responsible for most of the incretin effects, which in nondiabetic individuals is a normal physiological action. However, in T2DM patients the incretin effect is blunted: the so-called “incretin defect.” The “incretin defect,” a metabolic deterioration associated with T2DM, was demonstrated by Nauck et al. (1986) [84]. In their study, oral and intravenous glucose caused similar changes in plasma glucose concentration in subjects with T2DM. In healthy individuals, the insulin secretory response after oral glucose ingestion exceeded the response elicited by intravenous administration of an equal amount of glucose.

This “incretin defect” in T2DM seems to have two main causes [85]: reduced secretion of GLP-1 and intense impairment of the insulinotropic effect of GIP. In addition to the altered incretin effect, T2DM is also associated with defective release of GLP-1. Toft-Nielsen et al. studied incretin secretion, including GLP-1, within 4 hours after a meal in individuals with T2DM, and compared them with those who had a normal glucose tolerance [86]. The results showed a significant reduction of the GLP-1 response in patients with T2DM. In addition, in a small study of identical twins, differing only in their T2DM status, the GLP-1 response was reduced only in the diabetic twin [87].

Several observations suggest that the abnormal GLP-1 secretion is most likely a consequence rather than a cause of diabetes, including the study of Knop et al., which attempted to evaluate the reduced incretin effect as a cause or as a consequence, concluding that it is a characteristic consequence of the diabetic state rather than a primary event that leads to T2DM [88].

The pancreatic *β* cell mass of a normal person can adapt to different insulin requirements when challenged with different glucose loads. However, the ability of pancreatic *β* cells to release optimal and effective insulin may be compromised in diabetes. The inability of pancreatic *β* cells to balance insulin resistance is a major problem in patients with impaired glucose tolerance or overt T2DM. This defect is due to a structural lesion in the insulin molecule or its receptors. It may also be due to the inability of the endocrine pancreas to maintain optimal *β* cell mass capable of producing the required amount of effective insulin. Long-term T2DM puts a lot of stress on pancreatic *β* cells. The impact of a high workload and hyperglycaemia-induced oxidative stress can eventually lead to pancreatic *β* cell death.

Some authors have shown that incretin pathways play important roles in the progression of T2DM [20]. The significant reduction in the incretin effect seen in patients with T2DM has been attributed to several factors, including impaired secretion of GLP-1, accelerated metabolism of GLP-1 and GIP, and a defective responsiveness to both hormones [89]. While the GIP concentration is normal or modestly increased in patients with T2DM, its insulinotropic actions are significantly diminished. This implies that a defect exists at the physiologic or even supraphysiologic levels in patients with T2DM in response to GIP. The impaired responsiveness to GIP may suggest a possible link to GIPR downregulation or desensitization [1].

In contrast to GIP, the secretion of GLP-1 is reduced in obese subjects without diabetes, suggesting that incretin secretion is altered in the early stages of diabetes [21]. In patients with T2DM, the incretin effect is reduced or absent, which contributes to a defective first phase of insulin secretion [90]. Some authors report that the incretin effect is responsible for about 60% of the secretion of postprandial insulin, which is decreased in T2DM [91]. In these patients, GIP secretion is normal, but its insulinotropic effect is markedly reduced, while the GLP-1 secretion is reduced but preserves its insulinotropic action, meaning that it can still effectively stimulate insulin secretion [89]. The cause for the differing properties of the GIP and the GLP-1 incretin effect in relation to changes in T2DM is not fully understood. The finding that T2DM patients have low concentrations of GLP-1, but their response of insulin secretion is preserved, supports the therapeutic potential of GLP-1 treatments. Thus, while GIP has a low potential as a drug therapy, GLP-1, on the other hand, has a therapeutic potential as a promising pharmacological tool for the treatment of T2DM, already proposed in the 1990s, when the incretin effect was reviewed [92].

In contrast to other insulinotropic agents, such as sulphonylureas or glinides, the insulinotropic effect of GLP-1 depends strictly on glucose, providing the ability to normalize glucose values without the risk of hypoglycaemia, which is a quite relevant therapeutic approach. Furthermore, GLP-1 possesses a variety of additional physiological effects that are attractive in the treatment of T2DM, such as in the suppression of glucagon secretion from the pancreatic *α*-cells, in a glucose-dependent manner. This can represent an important advantage for those patients with hyperglucagonemia refractory to glucose administration, but that are still responsive to GLP-1 [20].

## 2. Type 2 Diabetes Therapeutics with Incretin Modulators

### 2.1. The Current Context of T2DM Pharmacotherapy

T2DM is a chronic disease with increasing prevalence in our society. Global estimates of the prevalence of diabetes for 2030 indicate a growing burden of the disease, particularly in developing countries, where a 69% increase in numbers between 2010 and 2030, ranging from 285 to 439 million adults (aged 20–79 years), was estimated [100]. About 60% of the patients who are diagnosed with T2DM do not achieve adequate glycaemic control and, therefore, have an increased risk for developing micro- and macrovascular complications. T2DM is the result of a complex array of metabolic abnormalities. The spectrum of metabolic alterations includes insulin resistance in muscle and liver, as well as a progressive *β* cell failure (the classic triad). Furthermore, from the triumvirate theory, Defronzo (2009) suggested there is much more to the pathogenesis of T2DM, suggesting five additional elements that make substantial contributions to the development and evolution of the disease: (1) alterations in the enteroendocrine physiology, (2) increased lipolysis in fat cells, (3) increased glucagon secretion, (4) increased renal reabsorption of glucose, and finally (5) CNS insulin resistance with appetite dysregulation [101]. Because of the interrelation of these 8 factors to the pathophysiology of T2DM and its associated morbidity and mortality, they have been referred to as the “ominous octet” [101].

These eight interrelated factors have important implications in the optimization of the treatment for patients with T2DM. First, it is because multiple abnormalities require the use of several drugs in order to correct the abnormal pathophysiology of T2DM. Second, treatment must address not only surrogate markers of the disease, such as elevated HbA1c levels, but also known pathogenic mechanisms. Finally, in order to prevent or slow progressive deterioration in *β* cell function, the interval between the beginning of T2DM and its diagnosis must be shortened so that treatment can be initiated as early as possible. In recognition of these important imperatives, treatment should include a combination of interventions.

T2DM treatment includes the “physiological” correction of insulin resistance and its defects in secretion. Therefore, besides lifestyle changes, especially diet and exercise, drug therapy is the basis of the treatment, including medication that reduces insulin resistance [such as biguanides or thiazolidinediones (TZDs)], insulin secretagogue agents [such as sulfonylureas (SU)], and/or insulin therapy in more advanced stages of the disease.

According to the main international institutions for diabetes care [American Diabetes Association (ADA), European Association for the Study of Diabetes (EASD), and International Diabetes Federation (IDF)], drug treatment in T2DM patients should be started when nutritional recommendations and physical activity are not effective to maintain HbA1c levels below 7.0%, even in patients without complications, with relatively “good” quality of life, and adhering to nutritional guidelines and physical activity [102, 103]. In patients with T2DM, the risk of complications is associated with the prior hyperglycaemic state, and any reduction in HbA1c levels promotes a reduction in the risk for complications [104]. Treatment regimens that reduce the levels of HbA1c to near or below 7% result in a significant reduction of risk of microvascular complications and diabetes-related death. Current recommendations by the Consensus of ADA and EASD justify the selection of appropriate treatment based on its ability to achieve and maintain glycaemic goals [102].

Table 1 features the main features of the antidiabetic armamentarium (non-incretin-based therapies), focusing on mechanisms of action, major effects/advantages, adverse reactions, and the ability to decrease HbA1c.

### 2.2. The Therapeutic Potential of Incretin Modulators in T2DM Management

The above discussion regarding the T2DM pathophysiology reasonably suggests that ideal antidiabetic therapy should address the “ominous octet.” Therefore, a drug (or a combination of drugs) that can ideally improve *β* cell health (TZDs, incretin-based therapies, biguanides, and *α*-glucosidase inhibitors), improve insulin resistance (biguanides, TZDs, and possibly incretin-based therapies), suppress glucagon secretion (incretin-based therapies), suppress appetite (GLP-1 analogues, biguanides), improve lipid health (TZD), and suppress renal glucose reabsorption causing the increase in urinary excretion (sodium-glucose cotransporter 2 inhibitors) would be the perfect therapy. Incretin-based therapies (addressing 4 out of the 8 pathophysiological defects) with biguanide [metformin (MET)] or TZD (addressing insulin resistance in liver and skeletal muscle) seem to be a very good option. Efforts should be made for restoring the GLP-1 physiologic function in T2DM and, thus, correct the multiple metabolic abnormalities observed in patients with the disease.

Following their secretion, GLP-1 and GIP are both rapidly degraded by DPP-4. This enzyme, originally described as a lymphocyte cell surface protein CD26, exists in two molecular forms with proteolytic activity: a membrane-spanning protein with a short intracellular tail and a soluble circulating protein, which lacks the intracellular tail and transmembrane regions [105]. The soluble form (sDPP-4) was first identified in serum and saliva and has been detected in cerebrospinal and seminal fluid and bile and accounts for a significant part of DPP-4 activity in human serum, as recently reviewed [106]. The transmembrane protein is expressed on many different cell types and tissues, including the gut, liver, spleen, lungs, brain, heart, endothelial capillaries, acinar cells of mucous and salivary glands, pancreas, uterus, and immune organs such as thymus, spleen, and lymph node, with the highest levels found in the kidney [107–109]. DPP-4 is the canonical representative of a family of genetically related peptidases. The DPP-4 gene family includes four enzymes DPP-4, DPP-8, DPP-9, and fibroblast activation protein (FAP), in addition to the catalytically inactive proteins DPP-6 and DPP-10.

DPP-4 regulates the activity of the secretory hormones GLP-1 and GIP to maintain glucose homeostasis (enhanced insulin secretion and glucagon suppression), thereby improving postprandial and fasting hyperglycaemia [110–112]. GLP-1 is degraded even before leaving the gut by DPP-4 molecules anchored to the luminal surface of endothelial cells of the mucosal capillaries. GIP is less susceptible to DPP-4 degradation and leaves the gut unchanged. DPP-4 has several other physiological substrates [including chemokines, neuropeptides, and regulatory peptides, such as neuropeptide Y (NPY), substance P, or stromal cell-derived factor 1 (SDF1)] and its expression is highly regulated, as recently reviewed [106].

Several studies have shown that circulating DPP-4 activity is increased [113–117] in diabetic patients and animals, but this finding is not consensual, as other studies reported decreased activity [118, 119].

In humans, circulating levels of intact GLP-1 decrease rapidly (half-life of about 2 minutes) due to inactivation by the DPP-4, such that biologically active GLP-1 represents only 10% to 20% of total plasma levels [17]. Therefore, strategies to increase GLP-1 levels in plasma are based on (a) the use of GLP-1 receptor agonists, such as exenatide, or GLP-1 analogues, resistant to enzymatic inactivation, such as liraglutide, collectively known as incretin mimetics or GLP-1 mimetics; (b) the use of DPP-4 inhibitors, also known as gliptins. Both agonists and analogues of GLP-1 have demonstrated their efficacy in the treatment of T2DM without causing hypoglycaemia but have the disadvantage of being injectable drugs. DPP-4 inhibitors, on the other hand, are orally active, but they may potentiate the action of other peptides that are also degraded by this enzyme. Together, these therapeutic strategies are called “incretin-based” therapies or “incretin modulators” and represented, in themselves, a promising development for the treatment of T2DM.

This review will now focus on DPP-4 inhibitors, exploiting their antidiabetic properties in comparison (and/or in association) with the preexisting oral antidiabetic agents arsenal, as well as the potential for acting as a cytoprotective agent in extrapancreatic organs/tissue, including some of those targeted by diabetic complication (heart, vessels, kidney, and retina). In sum, the place of gliptins in T2DM therapy is revisited, questioning if these agents are essentially identical to the previous hypoglycemic drugs (“*me too*”) or if they have potential to notably improve the management of diabetes and prevent its serious complications, thus acting as “*the special one*” antidiabetic drugs.

## 3. Dipeptidyl Peptidase-4 Inhibitors

In T2DM patients during hyperglycemic clamp studies, infusion of GLP-1, but not GIP, stimulates insulin secretion, thus showing that the insulinotropic effect of GLP-1 is reasonably well-preserved in T2DM, despite possibly lower levels, when compared to nondiabetic individuals [120, 121]. In contrast, despite relatively normal GIP levels, the insulinotropic effect of GIP is severely impaired, with the ability of GIP to stimulate second-phase insulin secretion being absent, although a first-phase response is present [120]. Hence, the development of incretin-based therapies for T2DM has thus far focused on GLP-1, rather than GIP. An important feature of GLP-1 is that it is rapidly secreted by the L-cells of the ileum, in just about 15 minutes after a meal, but it is also rapidly metabolized in the blood by DPP-4, becoming an inactive fragment. The extremely rapid and widespread degradation of GLP-1 by DPP-4 led to the proposal that enzyme inhibitors could be used therapeutically in T2DM, protecting and strengthening the circulating levels of GLP-1 [17, 122]. Human subjects with T2D exhibit relative resistance to the actions of GIP [19]. So, GIP is not an effective blood glucose-lowering agent in T2D subjects. As occurs with GLP-1, GIP is rapidly in vivo inactivated by DPP-4, converting full length GIP(1–42) to inactive GIP(3–42) within minutes of its secretion from the gut K-cell. Thus, one of the objectives of DPP-4 inhibition is to stabilize GIP [123], resulting therefore in greater insulinotropic activity [124], and to prolong the beneficial effects of endogenous GLP-1. The idea was promptly accepted by the pharmaceutical industry and many companies have embarked on the development of DPP-4 inhibitors for clinical use.

The first DPP-4 inhibitor to reach the market was sitagliptin, followed by vildagliptin and more recently by saxagliptin, alogliptin, and linagliptin [93–97]. Long-term studies with DPP-4 inhibitors in clinical development have shown a good safety profile, tolerance, and no immune adverse effects [122]. The first clinical studies were performed in 2001 by Ahrén et al. [125], when they confirmed a significant effect in reducing postprandial hyperglycaemia, by reducing fasting blood glucose levels and HbA1c values after oral administration of sitagliptin daily for 4 weeks in patients with T2DM [125]. In another study with the long-acting DPP-4 inhibitor, vildagliptin, a sustained effect on HbA1c was documented (1% reduction compared with placebo). This was a 52-week study where the existing treatment with metformin was accompanied by the DPP-4 inhibitor [126]. These first two DPP-4 inhibitors showed good oral bioavailability and a relatively long action, so that one daily administration results in the inhibition of DPP-4 in 70–90%, over a period of 24 hours, which is sufficient to fully protect the degradation of endogenous incretin hormones [127]. Clinically, both inhibitors were shown to have numerous advantages as they stimulate the synthesis of insulin, suppress glucagon secretion, lower levels of postprandial and fasting glucose, improve *β* cell function, and lower HbA1c values in T2DM patients [91, 128]. Sitagliptin and vildagliptin have significant antidiabetic effects even when administered alone, resulting in improved glycaemic control, which is further improved when given in combination with other antidiabetic agents, including metformin, sulfonylureas, and thiazolidinediones [127]. In contrast to incretin mimetics, the DPP-4 inhibitors do not cause a reduction in body weight.

Although various DPP-4 inhibitors have different pharmacokinetic and pharmodynamic profiles, they are remarkably similar with regard to their antihyperglycaemic properties with a very safe profile (neutral concerning weight, without causing hypoglycaemia). These agents are all low-molecular-weight compounds, although they differ widely in terms of their chemical structure. Chemical DPP-4 inhibitors have been mainly divided into two series/families: peptidomimetics and nonpeptidomimetics compounds [129]. Vildagliptin and saxagliptin are peptide-like compounds based on a dipeptide structure, whereas other DPP-4 inhibitors are nonpeptidomimetic substances with an ample chemical diversity, including b-amino acid-based compounds (sitagliptin), modified pyrimidinediones (alogliptin), and xanthines (linagliptin). The nonpeptidomimetics compounds might assume special relevance because they show selectivity for DPP-4 versus other members of the DPP-4-like family of proteases, including DPP-2, DPP-8, and DPP-9, thus avoiding interference with other putatively important functions (although the in vivo functions remain largely unknown), as well as the possibility of undesirable side-effects [130].

The pharmacokinetic profile and the clinical features of DPP-4 inhibitors have been reviewed in the last years [22, 98, 131–133], and the main features of the class, as well as of each of individual drugs, are summarized in the following subtitles and in Tables 2 and 3, respectively.

### 3.1. Sitagliptin

Sitagliptin, from Merck Sharp & Dohme, was the first selective DPP-4 inhibitor in the market, approved in 2006 by FDA and commercialized as Januvia. Sitagliptin promotes around 97% of DPP-4 inhibition [98] and reduces blood glucose levels, either in the postprandial or the fasting state. It works differently from other drugs already available for diabetes and it is orally active [134]. It presents a bioavailability of 87% and can be taken with or without food, with a recommended dose of 100 mg once daily in the EU and in the USA. The hepatic metabolism of sitagliptin is minimal (mainly by cytochrome P450 3A4) and it is largely (70–80%) excreted by the urine in its unchanged form, with a half-life of around 12 hours [135]. As a result of its metabolism and elimination, dose adjustment is required in patients with severe renal impairment, but not in those with mild or moderate renal or hepatic impairment [136, 137]. No dosage adjustment is necessary on the basis of age, gender, race, or body mass index; in addition, sitagliptin has a low propensity for pharmacokinetic drug interactions [93].

Randomized placebo- or active comparator-controlled trials have demonstrated the efficacy of sitagliptin in terms of improving glycaemic control in T2DM patients, used as monotherapy, initial combination therapy (usually with fixed-dose combinations of sitagliptin/metformin), or add-on therapy to metformin or to other antihyperglycaemic drugs, with or without metformin. Sitagliptin showed efficacy in decreasing HbA1c, fasting plasma glucose (FPG), and 2 h postprandial glucose (PPG) levels, also increasing the proportion of patients achieving target HbA1c levels.

Several randomized, double-blind, placebo-controlled trials with sitagliptin as monotherapy in adult patients with T2DM and inadequate glycaemic control (HbA1c typically 7–10%) showed statistically significant placebo-corrected reductions from baseline HbA1c (0.6–1.1%), in FPG (1.0–1.8 mmol/L) and in 2-h PPG (2.6–4.5 mmol/L; *p* < 0.01), among patients receiving sitagliptin. In addition, the proportion of patients achieving target HbA1c levels (<7.0%) at the end of the study period was significantly (*p* < 0.001) superior among sitagliptin-treated patients (21–58%) compared to placebo recipients (5–17%) [138–142]. The results of randomized, double-blind, active comparator-controlled trials showed noninferiority of sitagliptin monotherapy versus metformin and versus voglibose in patients with normal renal function and noninferiority versus glipizide in patients with renal impairment [143–145].

Several randomized, double-blind trials in T2DM adults inadequately controlled with diet and exercise showed improved glycaemic control from initial combination therapy with sitagliptin and other antihyperglycaemic agents, such as those with the biguanide metformin in fixed-dose combination formulations and those with the peroxisome proliferator-activated receptor-*γ* (PPAR*γ*) agonist pioglitazone (thiazolidinedione). Initial combination sitagliptin (50 mg)/metformin (500 mg) twice daily or 50 mg/1000 mg twice daily achieved significantly greater reductions in HbA1c and FPG levels when compared to corresponding total daily dosages of sitagliptin or metformin monotherapy, and significantly more patients receiving combination therapy achieved target HbA1c levels of <7.0% [146–151]. Combination therapy with pioglitazone also had significantly greater effects on reductions from baseline HbA1c levels than with pioglitazone monotherapy and improvement of other glycaemic parameters, including FGP reductions, together with a significantly greater proportion of patients achieving target HbA1c levels [152–154], despite being less important than combination with metformin (57.3 versus 43.5%) [151].

A number of large randomized, double-blind, placebo-controlled [155–158], and active-comparator [159–165] trials have evaluated the efficacy of sitagliptin as add-on therapy to metformin (>1500 mg/day) in adults with inadequately controlled T2DM. Addition of sitagliptin (50 [156] or 100 [155, 157, 158] mg/day) to ongoing metformin for 12–24 weeks was superior to placebo plus metformin in reducing placebo-corrected HbA1c (0.65–1.0%), FPG (1.0–1.4 mmol/L), and 2 h PPG (1.9–3.0 mmol/L), with a greater proportion of patients achieving the target HbA1c levels: 14–47% for sitagliptin versus 3–18% placebo, across all four studies [155–158]. Various randomized studies of 18–52 weeks' duration compared the efficacy of sitagliptin (100 mg/day) as add-on therapy to metformin with that of other orally administered antihyperglycaemic agents, including sulfonylureas (glimepiride and glipizide) and PPAR*γ* agonists (pioglitazone and rosiglitazone). Two large studies comparing sitagliptin with sulfonylureas as add-on therapy to metformin demonstrated noninferiority between treatment groups [158, 162]. The addition of sitagliptin to ongoing metformin achieved reductions in HbA1c broadly similar to those observed when pioglitazone 45 mg/day or rosiglitazone 8 mg/day was added to metformin for 26 or 18 weeks, respectively [161, 165]. Other large randomized, placebo-controlled trials have evaluated the efficacy of sitagliptin as add-on therapy to ongoing treatment with a PPAR*γ* agonist, a sulfonylurea, or insulin, with or without metformin, and the results, overall, showed that patients randomized to sitagliptin had statistically significant placebo-adjusted reductions of HbA1c (0.6–0.9%), FPG (0.8–1.1 mmol/L), and 2-h PPG (2.0–2.7 mmol/L), as well as a great proportion of patients that achieved target HbA1c (13–45% versus 3–23%) [166–172].

Sitagliptin has a neutral effect on body weight, as reported in almost all of the studies previously mentioned [138–142]. Concerning the impact of sitagliptin on lipid profile, the available data showed no consistency, but the majority of studies reported a beneficial effect on TGs, HDL-c, and LDL-c, as concluded in a systematic review and meta-analysis of 14 trials with incretin therapy in patients with T2DM [173]. In addition, some studies suggested a reduction of blood pressure in patients under sitagliptin treatment [174–176]. The reduction of global cardiovascular risk factors by sitagliptin seems to be important for improving outcomes in patients with T2DM [177].

Sitagliptin is well tolerated and the risk of adverse events, including hypoglycaemia, is very low [132, 147, 155, 162]. The most common are GI disturbances, including abdominal pain, nausea, vomiting, and diarrhoea, which are rare when used in monotherapy [67]. Nasopharyngitis, upper respiratory tract infections, and headache occur in a low percentage of patients (versus placebo) [173]. The prescribing information for sitagliptin includes information regarding postmarketing reports of acute pancreatitis, but the association between DPP-4 inhibitor use and pancreatitis remains controversial, as further discussed.

### 3.2. Vildagliptin

Vildagliptin, from Novartis, commercialized firstly with the brand name of Galvus, is a highly selective, reversible, inhibitor of the enzyme DPP-4 approved by the EU in 2007 for the treatment of T2DM, with a recommended dose of 50 mg twice daily. Vildagliptin treatment results in a rapid inhibition of DPP-4 (around 95% of maximal inhibition [98]), causing elevation of endogenous levels in fasting and postprandial incretin hormones, GLP-1 and GIP, thus improving *β* cell sensitivity to glucose and resulting in the increased secretion of glucose-dependent insulin. Furthermore, vildagliptin also enhances the sensitivity of *α* cells to glucose, resulting in an improvement in the homeostasis of glucagon. Oral vildagliptin had an absolute bioavailability of about 85% and can be administered with or without food, although food slightly delayed the *t*_max⁡_ and decreased *C*_max⁡_ by about 20% [94]. Vildagliptin is not metabolized by cytochrome P450 enzymes to a quantifiable extent and, thus, it is unlikely to affect the metabolism of other drugs or to be affected by them. The major metabolite (carboxylic acid) is obtained by hydrolysis and is pharmacologically inactive, the kidney being responsible for the hydrolysis and for excretion (about 55% as unchanged parent and about 26% as metabolite) [94].

The efficacy of vildagliptin monotherapy with that of placebo in patients with T2DM was analyzed in randomized, double-blind, multicentre trials of 12, 24, or 52 weeks' duration. Vildagliptin improved glycaemic control, viewed by reduction of HbA1c, which was usually more effective for higher basal levels [178–181]. In addition, FPG levels were also significantly reduced by vildagliptin versus placebo [178–180]. The efficacy of vildagliptin monotherapy was also compared with that of other oral antihyperglycaemic agents, examining noninferiority of vildagliptin with the comparator. After 12 weeks' therapy, a significantly greater reduction from baseline in HbA1c was seen with vildagliptin than with voglibose [182]. After 24 or 104 weeks' therapy, the proportion of patients achieving the target HbA1c of <7.0% did not significantly differ between patients receiving vildagliptin and those receiving gliclazide [183] or acarbose [184], while when compared with metformin the percentages were 35% with vildagliptin versus 45% with metformin [185]. Significantly more vildagliptin than voglibose recipients achieved an HbA1c of <6.5% [182]. Once again, the reduction of HbA1c tended to be higher in patients with higher baseline levels [182–186]. In addition, in vildagliptin recipients a significant reduction from baseline in FPG levels was seen; however, the mean reduction was significantly higher with metformin, rosiglitazone, or gliclazide than with vildagliptin, and noninferiority between vildagliptin and acarbose was not established [182–186]. On the contrary, reduction from baseline in FPG levels was significantly greater with vildagliptin than with voglibose [182].

The efficacy of vildagliptin administered in combination with metformin in the treatment of T2DM patients was evaluated in randomized, double-blind, multicentre trials of 12, 24, and 52 weeks' duration. Combination therapy with vildagliptin 50 mg twice daily plus metformin, in 24-week trials, improved HbA1c to a significantly greater extent than monotherapy with metformin or vildagliptin in patients with T2DM poorly controlled by metformin monotherapy or who were treatment naïve [187, 188]; patients receiving vildagliptin plus metformin showed reduced HbA1c levels until week 12 thereafter and remained stable [187, 188]. A greater proportion of patients receiving vildagliptin (50 mg twice daily) plus metformin (500 or 1000 mg twice daily) than vildagliptin or metformin monotherapy have achieved the target HbA1c levels of <7% (55.4% and 65.4% versus 40.0% and 43.5%), the reductions being higher for higher baseline levels [188]. In addition, FPG levels were also reduced to a significantly greater extent with vildagliptin combined with metformin than monotherapies [187, 188]. Vildagliptin plus metformin demonstrated noninferiority to pioglitazone plus metformin concerning change in HbA1c after 24 weeks in T2DM patients inadequately controlled by metformin monotherapy [189]. Regarding change from baseline in FPG, noninferiority of vildagliptin plus metformin versus pioglitazone plus metformin was not established after 24 weeks' therapy [189]. Following the 28-week single-blind phase, the mean change from baseline in HbA1c at week 52 was identical (−0.6%) in both patients receiving vildagliptin plus metformin and those receiving pioglitazone plus metformin [190].

The results of two 52-week trials showed noninferiority (in terms of the change from baseline in HbA1c) of vildagliptin plus metformin combination therapy when compared with glimepiride or gliclazide plus metformin [191, 192]; the proportion of patients achieving an HbA1c of <7.0% was 29.6% and 54.1% with vildagliptin plus metformin, 31.9% with gliclazide plus metformin, and 55.5% with glimepiride plus metformin [191, 192]. The reductions in HbA1c in the vildagliptin plus metformin recipients tend to be higher in those with higher baseline HbA1c levels [191, 192]. Noninferiority, in terms of the change from baseline in FPG, was demonstrated in the vildagliptin plus metformin combination versus gliclazide plus metformin and there were no differences versus glimepiride plus metformin [191, 192].

The efficacy of vildagliptin administered in combination with pioglitazone or glimepiride was evaluated in randomized, double-blind, multicentre trials of 12 or 24 weeks' duration, with T2DM patients who had not received pharmacological therapy for at least 12 weeks or who were inadequately controlled with thiazolidinedione or sulfonylurea monotherapy. The combination of vildagliptin (50 mg twice daily) with pioglitazone or with glimepiride significantly improved the glycaemic control, after 34 weeks, viewed by greater reductions in HbA1c versus monotherapy with pioglitazone or with glimepiride [193, 194], and a significantly greater proportion of patients receiving combination with pioglitazone versus pioglitazone alone (36.4% versus 14.8%) and with glimepiride versus glimepiride alone (21% versus 12%) achieved an HbA1c of <7% [193, 194]. Once again, the reductions were higher for those patients with higher baseline HbA1c levels. No differences were encountered for change from baseline in FPG between combined therapies versus monotherapies after 24 weeks' therapy [193, 194].

Vildagliptin has been associated with low incidence of adverse reactions, including hypoglycaemia, and is neutral in terms of effects on body weight [173]. No reactions were found to be associated with age, ethnicity, duration of exposure, or daily dose of the drug. The majority of the adverse reactions in the various studies were mild and transient, not requiring discontinuation of treatment. Concerning vildagliptin monotherapy, at a dose of 100 mg per day, the adverse reactions reported, beyond those observed in patients receiving placebo, were dizziness, headache, peripheral oedema, constipation, nasopharyngitis, upper respiratory tract infection, and arthralgia [178–181]. The use of vildagliptin in patients with moderate-to-severe renal or hepatic insufficiency is not recommended, and there is a requirement for liver enzyme monitoring to avoid possible hepatic adverse events [98].

### 3.3. Saxagliptin, Alogliptin, Linagliptin, and Other DPP-4 Inhibitors

Saxagliptin, from Bristol-Myers Squibb, was approved by the FDA in 2009 and marketed as Onglyza and is another DPP-4 potent inhibitor with pharmacokinetic and pharmacodynamic properties suitable for once-daily dosing, with or without food, at any time of the day [195, 196]. Saxagliptin has a maximal inhibition of DPP-4 of around 95% [98] and is metabolized hepatically by cytochrome P450 (CYP) 3A4/5 to an active metabolite, 5-hydroxy saxagliptin, which is also a selective, reversible, competitive DPP-4 inhibitor, but it is half as potent as the parent compound [197]. The half-life after a single oral dose of 5 mg/day, the recommended dose, is 2.5 h for saxagliptin and 3.1 h for the active metabolite; the elimination of saxagliptin is both hepatic and renal in the parental form or as metabolite [98]. Dose adjustments (reduction to 2.5 mg/day) are recommended in patients with moderate-to-severe renal impairment since systemic exposure to the drug increases in proportion to the degree of renal impairment [198]; renal function should be assessed prior to initiating saxagliptin therapy and monitored periodically thereafter; its use is presently not recommended in patients with severe hepatic insufficiency [98].

Saxagliptin is approved as a combination therapy with metformin, sulfonylurea, thiazolidinedione, or insulin (with or without metformin) to improve glycaemic control in adult patients with T2DM who do not achieve adequate glycaemia control with metformin, sulfonylurea, thiazolidinedione, or insulin in addition to diet and exercise, including patients with mild-to-severe renal impairment. The efficacy of saxagliptin as add-on therapy to various baseline antihyperglycaemic agents has been demonstrated in a number of clinical trials of 18 to 104 weeks' duration. In combination with metformin, glibenclamide, thiazolidinedione, or insulin (with or without metformin), saxagliptin was significantly more effective than placebo in lowering HbA1c, FPG, and PPG levels, as previously reviewed [95].

Similar to other DPP-4 inhibitors, pooled data from saxagliptin monotherapy and combination therapy trials demonstrate that saxagliptin is generally well tolerated, with a very low risk of adverse events, including hypoglycaemia, and is generally weight neutral; current prescribing information contains a warning regarding postmarketing reports of pancreatitis [199, 200].

Alogliptin was approved by FDA in 2013 and is marketed by Takeda Pharmaceutical Company as Nesina. It is a highly selective DPP-4 inhibitor, with a maximal inhibition of >90%; the hepatic metabolism, mediated by cytochrome P450 (CYP) 2D6, is minimal and it is largely (60–70%) excreted by the urine in its unchanged form; its half-life varies between 11 and 22 h [96, 98]. The pharmacokinetic properties of alogliptin did not alter to any clinically significant extent based on age, race, or sex.

The recommended dose is 25 mg once a day. In several large trials of up to 26 weeks' duration, alogliptin in monotherapy or in combination with other oral antihyperglycaemic agents (metformin, glibenclamide, or pioglitazone) or insulin therapy has improved glycaemic control in adult patients with inadequately controlled T2DM [201, 202]. As reported for the other drugs of this class, alogliptin is well tolerated, including elderly patients, and the incidence of hypoglycaemia is lower, with neutral effects on body weight and lipid parameters. Considering the primarily renal elimination, alogliptin treatment should be accompanied by dose adjustment in patients with moderate-to-severe renal impairment.

Linagliptin was approved in 2011 by FDA (marketed as Trajenta by Eli Lilly Co. and Boehringer Ingelheim) and is a xanthine derivative with singular pharmacokinetic properties when compared with previously commercialized DPP-4 inhibitors, which may offer some advantages in clinical practice [97, 203]. Therefore, at recommended therapeutic doses (5 mg once a day), linagliptin has a low oral bioavailability (±30%), but a large apparent volume of distribution, demonstrating extensive distribution into tissues; it has a long half-life (>100 h), due to its extensive binding to plasma proteins and its high-affinity binding to the DPP-4 enzyme, which produces a nonlinear pharmacokinetic profile. The nonlinear pharmacokinetics of linagliptin are best described by a two-compartmental model that incorporates target-mediated drug disposition resulting from high-affinity, saturable binding to DPP-4 [204]. The strong link to DPP-4 (which is inhibited in >90%) and the capacity to dissociate at a very low velocity prolong the in vivo action, allowing a once-a-day administration, thus improving compliance [205]. A major pharmacokinetic property is the nonrenal elimination route, which allows its use in patients with renal impairment without dose adjustment or monitoring of renal function. In fact, linagliptin is poorly metabolized and mainly eliminated by biliary rout, with a very small renal elimination (<6%), which might explain the possibility of using Linagliptin in renal insufficiency patients [98, 206, 207], which is unique when compared to sitagliptin and saxagliptin, both requiring renal dose adjustment. Despite the predominantly hepatic elimination, the main metabolite (CD1790) is pharmacologically inactive, and no adjustments are currently recommended in patients with hepatic impairment. In addition, no meaningful impact of age, sex, or race on the pharmacokinetic properties of linagliptin has been observed.

The efficacy of linagliptin is similar to that of the DPP-4 inhibitors previously discussed [208], when used as monotherapy, initial combination therapy (with metformin or pioglitazone), or add-on therapy to other oral antihyperglycemic agents (metformin and/or sulfonylurea) or basal insulin (with or without metformin and/or pioglitazone), improving the glycaemic control parameters, with a mean HbA1c reduction between 0.5 and 0.7% [97, 203]. Data pooled from randomized, double-blind, placebo-controlled clinical trials lasting ≤24 weeks shows that linagliptin is well tolerated, with a low risk of hypoglycaemia, and is weight neutral [209]. The efficacy of linagliptin may be limited in patients receiving concurrent inducers of CYP3A4 or P-gp (e.g., rifampin). A risk for hypoglycemia might exist when linagliptin, as well as another DPP-4 inhibitor, is used as a treatment adjunctive to an insulin secretagogue, and an initial dose decrease in background secretagogue medication should be considered to prevent hypoglycemic events.

Many other DPP-4 inhibitors have been developed and commercialized (namely, in Japan) or are yet under clinical trials. Anagliptin and Teneligliptin were both already approved in Japan in 2012, while other agents (such as Gemigliptin and Dutogliptin) are yet in clinical trials or initiating approval procedures, mainly in Asia countries.

## 4. Major Challenges and Future Prospects

### 4.1. The Place of Gliptins in T2DM Therapeutics in the Future

DPP-4 inhibitors undoubtedly constitute an innovative class of oral agents for the treatment of T2DM which have enlarged the therapeutic possibilities. The main mechanism of action of DPP-4 inhibitors is essential to keep endogenous GLP-1 from being degraded, by inhibiting DPP-4. Current indications for DPP-4 inhibitors recommend its use in combination with other antidiabetic agents, in particular with metformin, as second and third line therapy, and even over other antidiabetic therapies, especially if the patient is experiencing an increased incidence of hypoglycaemia.

Considering the above described characteristics of DPP-4 inhibitors, they could revolutionize the concept of diabetes management, either alone or in combination with other antidiabetic drugs. However, there are currently still some questions for which there is no complete answer: whether DPP-4 inhibitors can promote preservation of human *β* cell function; the most proper stage of disease to initiate therapy; and the long-term safety of gliptins. T2DM is characterized by a progressive loss of *β* cells mass and function that is associated with insulin resistance. These defects are followed by a significant decrease in the incretin effect, which are mainly related to abnormalities in GLP-1 secretion and action. Since DPP-4 inhibitors are dependent on the endogenous secretion of incretins, that class of drugs will theoretically be useful in early stages of diabetes, when the patient still retains a *β* cell population capable of responding to GLP-1 stimulation. In fact, the possibility of using incretin modulators, including (but not only) DPP-4 inhibitors, in prediabetes is also under debate. On the other hand, and according to their benefit in reducing the levels of glucagon, DPP-4 inhibitors can also be used in the later stages of the disease, in combination with other oral antidiabetic agents, in poorly controlled patients, as is the current clinical indication. Furthermore, the use of incretin modulators (including DPP-4 inhibitors) in conjugation with insulin in later stages of the disease has been extensively discussed during the last years. The safety and tolerability of DPP-4 inhibitors seem to be generally comparable to nongliptin treatments, although long-term studies and clinical practice followup are still needed, in particular to definitively evaluate if there is any reasonable association between the use of these agents and the risk of pancreatitis and pancreatic cancer, as suggested by some reports.

Table 2 provides a sum-up of DPP-4 inhibitors in terms of their mechanism of action, major biological effects/advantages, adverse reactions, and their ability to decrease HbA1c, which can be compared with Table 1, which summarizes the same properties for the other (non-incretin-based) oral antidiabetic agents.

In addition, the possibility of cytoprotective properties afforded by DPP-4 inhibitors on organs/tissues which are affected by diabetes (such as the heart, vessels, kidneys, and retina) and associated with serious diabetic complications might open new avenues for the use of these agents in the treatment of diabetic patients. The main challenges described above will be briefly revisited in the following subtitles.

### 4.2. Unanswered Questions and Evolving Issues

#### 4.2.1. Risk of Pancreatitis

During the last years, several lines of evidences indicate that GLP-1-based therapies could cause pancreatitis or pancreatic cancer [210, 211]. These suggestions came from few preclinical studies [212–214], which are basically inconclusive because the histological changes are not reproduced in all studies and vary between different GLP-1-based therapies and from very limited clinical data [215, 216], namely, observational studies [217–221], and from pancreases from organ donors with and without diabetes [222], which have limited value to conclude the issue, as commented by Ryder (2013) [223]. In addition, the increased reports of pancreatitis and pancreatic cancer by the FDA adverse event reporting system for exenatide and sitagliptin [224] are prone to bias, probably associated publicity surrounding these issues, and are thus not useful for establishing the incidence of adverse events. For that reason, it is decisively important to have data from well-controlled long-term studies, which are still lacking.

The strongest evidences currently available come from two large cardiovascular safety studies with DPP-4 inhibitors and from meta-analysis recently published of nonrandomized and randomized clinical trials. The SAVOR-TIMI 53 (saxagliptin) and EXAMINE (alogliptin) trials enrolled 16,492 and 5,380 patients over a median of 2.1 and 1.5 years, respectively, and concluded that there was no difference between the DPP-4 inhibitor treated and placebo groups with regard to pancreatitis or pancreatic cancer [225, 226]. A meta-analysis of 53 randomized clinical trials (including 20,312 patients treated with different DPP-4 inhibitors) did not find an increased risk of pancreatitis in DPP-4-treated patients [227]. Identical conclusion was achieved by the analysis of 19 RCTs comprising 10,246 patients treated for up to 2 years with sitagliptin [228]. Finally, Li et al. (2014) recently reviewed the data concerning the risk of pancreatitis in patients with T2DM under incretin-based therapies, by analyzing 60 studies (*n* = 353,639), consisting of 55 randomised controlled trials (*n* = 33,350) and five observational studies (three retrospective cohort studies and two case-control studies; *n* = 320,289), concluding that these drugs do not increase the risk of pancreatitis [229]. In addition, FDA and EMA independently evaluated the safety data from postmarketing reports of pancreatitis and pancreatic cancer in patients using incretin-based drugs, analysing both animal and clinical information available, and concluded that a causal association between incretin-based drugs and pancreatitis or pancreatic cancer cannot be established with the current data; however, FDA and the EMA have not reached a final conclusion regarding such a causal relationship, and both agencies will continue to investigate the safety signal [230].

So, at this stage, we should recognize that the link between these therapies and proven clinical pancreatitis and pancreatic cancer is not established. However, current evidence is not definitive and we should undoubtedly remain vigilant about the possibility of an association between GLP-1-based therapies and pancreatic disease and more carefully designed and conducted studies are warranted to definitively conclude this issue.

#### 4.2.2. Gliptins in Prediabetes

Prediabetes has been defined as a state of impaired fasting glucose (IFG) concentration ranging between 110 and 126 mg/dL [for the World Health Organization (WHO)] or between 100 and 125 mg/dL (for the American Diabetes Association (ADA)) and/or impaired glucose tolerance (IGT), characterized by a plasma glucose concentration 2 h after 75 g oral glucose load ranging between 140 and 199 mg/dL [231, 232].

It is widely accepted that insulin resistance starts several years before the onset of diabetes and *β* cell dysfunction is already present, even in the prediabetic stage. Several pathophysiological mechanisms contribute to the evolution of T2DM, including increased insulin resistance in the skeletal muscle and liver; augmented hepatic glucose output; impaired insulin secretion with progressive decline of pancreatic *β* cell function. Chronic hyperglycaemia and increased free fatty acids cause glucotoxicity and lipotoxicity, which accelerates *β* cell failure by apoptosis and decreased proliferation. In addition, deficiency of incretin secretion by the GI tract and/or resistance to incretin action due to downregulation of their receptors have been associated with evolution of diabetes [233]. Since GLP-1 is an insulin secretagogue and a suppressor of glucagon secretion, defects in GLP-1 secretion could contribute to the pathogenesis of prediabetes [234]. In fact, recent reports show that prediabetic patients with impaired glucose tolerance (IGT) and insulin resistance have decreased GLP-1 concentrations and early glucagon suppression [235, 236].

Considering the progressive decline of incretin effect in T2DM patients and the beneficial effects of incretin modulators in the treatment of diabetes, their use has been extended to patients with prediabetes, in a few recent small studies, as reviewed by Ahmadieh and Azar (2014) [237]. Particularly, the putative preservation of *β* cell function and mass afforded by these agents, in animal studies and in clinical trials, might help maintain good long-term metabolic control. However, the very small clinical experience on the use of DPP-4 inhibitors and GLP-1 mimetics in individuals with impaired fasting glucose or impaired glucose tolerance and the unsolved aspects related to the possibility of pancreatic side-effects do not recommend incretin-based therapies as an option for treatment in patients with prediabetes.

#### 4.2.3. Insulin Plus DPP-4 Inhibitors

Although incretin therapy has been mainly used in combination with oral antihyperglycemic agents, especially metformin, the potential use in association with insulin has been debated and increasingly tested during the last years. The complementary actions of the two approaches offer a promising strategy as a glucose-lowering treatment for T2DM patients, as recently revised by Vora et al. (2013) and Ahrén (2014) [238, 239]. In fact, there are several benefits of combining incretin-based therapies with insulin therapy, including the lowering of HbA1c due to combined reduction of fasting plasma glucose (FPG) by insulin and postprandial glycemia by incretins; reduction of risk of hypoglycemia which is due to the protection against hypoglycemia with incretin therapy in association with the often observed reduction in insulin dose when using this combination; the lower risk for weight gain given the protection afforded by incretin therapy, thus compensating the possible weight gain evoked by insulin therapy; the potential for long-term disease modifying effects, namely, by *β* cell function protection by insulin due to normalization of fasting glucose and prevention of glucotoxicity, combined with beta cell protection afforded by GLP-1-based therapies.

Several clinical studies have been reinforcing the possibility of a beneficial combination between DPP-4 inhibitors and insulin therapy. Several studies have analyzed the impact of adding DPP-4 inhibitor, during at least 24 weeks, on patients ongoing insulin therapy (alone or with metformin) with an insufficient glycemic control. Despite some minor variations between DPP-4 inhibitors (sitagliptin, vildagliptin, alogliptin, saxagliptin, and linagliptin), the combination treatment group (versus the placebo arm) showed a higher reduction of HbA1c (change between −0.5 and −0.8) and FPG (change between −0.2 and −1.0), as well as a reduced risk of hypoglycemia and weight gain [240–248]. There was no evidence of additional concern for safety or tolerability by combining incretin therapy and insulin in these studies.

Further studies, comparing different DPP-4 inhibitors and distinct insulin therapies, with comparable protocols and cohorts, will be very important to clarify the long-term efficacy and safety of these combinations. The current knowledge indicates that the combination is a very promising glucose-lowering strategy for the treatment of T2DM in patients who do need intensified therapy to control glycaemic levels.

### 4.3. Protective Effects of DPP-4 Inhibitors on Extrapancreatic Organs/Tissues

Since incretin hormones response is typically blunted in patients with T2DM, selective inhibition of DPP-4 can prolong their antihyperglycemic effects by increasing their circulating lifetime [249]. Furthermore, not only GIP and GLP-1 levels are affected by the modulation of DPP-4 activity, but also many other substrates, with a wide variety of physiological functions, can be modified, suggesting that DPP-4 inhibitors may participate in events other than the increase of incretin levels and glycemic control. Although a number of recent experimental studies have demonstrated beneficial effects of incretin-based therapies in extrapancreatic organs or tissues, including the vasculature [116, 250, 251], the kidney [252], the heart [253], and the brain [254], it remains unclear whether these effects are direct or mediated by the improvement of the glycemic control. It is also poorly understood whether those findings are observed in humans, indicating that further research is warranted to confirm the results of the preclinical studies.

#### 4.3.1. Sitagliptin

During the last years, our group has been studying the putative beneficial effects of DPP-4 inhibition with sitagliptin on several tissues in animal models of T2DM and T1DM. A therapeutic low dose of sitagliptin, during a 6-week treatment, in an animal model of T2DM, the Zucker Diabetic Fatty (ZDF) rat, was able to promote a partial correction of glycaemia and HbA1c levels when compared to controls, accompanied by a partial prevention of insulinopenia [255]. Furthermore, the ZDF rats treated with sitagliptin showed reduced blood pressure, total cholesterol, and TGs levels, suggesting possible cardioprotective effects. In addition, this DPP-4 inhibitor also showed a positive impact on low-grade inflammation, with decreased serum hsCRP levels, as well as an improvement in the redox status, which was accompanied by reduction of heart, pancreas, and kidney lipid peroxidation [255]. Moreover, sitagliptin treatment ameliorated both endocrine and exocrine pancreas lesions, as well as the glomerular, tubulointerstitial, and vascular lesions, together with a decrease in urea levels [252].

Besides the insulinotropic effects of GLP-1R activation in pancreatic cells, the expression of this receptor in a wide range of tissues, including retina [256, 257] and kidney [258], suggests the possibility of extrapancreatic effects. In fact, we observed that sitagliptin induced an increase in the levels of renal GLP-1 and its colocalization with GLP-1R in kidney tissue of diabetic ZDF rats, suggesting that GLP-1 may exert cytoprotective effects as we found an improvement of renal lesions, including glomerular, tubulointerstitial, and vascular lesions [252], as well as prevention of inflammation and apoptosis induced by diabetes [258]. In another experimental study, Abd El Motteleb and Elshazly (2013) described a protective effect of sitagliptin against L-NAME induced hypertensive nephropathy, related to increased levels of GLP-1, upregulation of GLP-1R, and consequent overexpression of eNOS and increased serum NO levels, together with improvement of redox status [259].

Although renoprotective properties of DPP-4 inhibitors have been suggested during the last years [109, 260–263], namely, based on experimental data, few studies have been performed in humans to assess the effects of DPP-4 inhibitors on kidney function metrics. Hattori (2011) evaluated the effect of sitagliptin (50 mg/day) on albuminuria in T2DM patients and found a significant decrease in HbA1c, FPG, and PPG, as well as in glycated albumin after 3 and 6 months [264]. Significant reductions in hsCRP and soluble vascular cell adhesion molecule 1 were also observed at 6 months. These authors also found that urinary albumin excretion (measured as urinary albumin-to-creatinine ratio) did not change in the 6 months before sitagliptin treatment and decreased in the 6 months after sitagliptin treatment, suggesting that sitagliptin lowered albuminuria without decreasing the estimated glomerular filtration rate. These effects seem to be related to blood glucose, inflammation reduction, or even increased levels of active GLP-1 [264]. Recently, Mori et al. (2014), in an open-labelled, prospective randomized study, evaluated the effects of 50 mg/day of sitagliptin (versus other oral glucose-lowering agents) on urinary albumin excretion in T2DM patients, during 6 weeks. Though both of the treatments significantly reduced HbA1c and FPG level, only sitagliptin significantly reduced urine albumin excretion, suggesting effects independent of the glucose-lowering effect of sitagliptin [265].

A recent retrospective study performed for 2 years in T2DM that aimed to determine the hypoglycemic effect of 2 years of sitagliptin administration revealed that the HbA1c levels decreased and C-peptide immunoreactivity (CPR) index increased from baseline to 3, 6, 12, 18, and 24 months after sitagliptin initiation [266], suggesting that sitagliptin improves glycemic control via an improved intrinsic insulin response.

Only few experimental studies have assessed the beneficial effect of sitagliptin in diabetic retinopathy. Our group has shown that sitagliptin induced a reduction in the nitrosative stress and inhibited inflammation and apoptosis of retinal cells in the ZDF rat retinas [256]. In a recent work, our group has also reported that sitagliptin prevented the diabetes-induced increase in DPP-4/CD26 activity and levels in serum and retina of streptozotocin- (STZ-) induced T1DM rats. Furthermore, sitagliptin prevented the increase in blood-retinal barrier permeability and decreased the retinal inflammatory state and neuronal apoptosis, thus indicating that it has direct protective effects on the retina that are independent of its antihyperglycemic effects [116].

There is growing evidence in the literature demonstrating the beneficial effects of sitagliptin on myocardial injury and cardiac function [267–269]. Picatoste et al. (2013) have shown that treatment of T2DM Goto-Kakizaki rats with sitagliptin (10 mg/kg/day) for 10 weeks promoted GLP-1-mediated cardioprotection primarily by limiting hyperglycaemia and hyperlipidemia [269]. In another study, treatment with sitagliptin (300 mg/kg/day) for 2 weeks in T1DM Fischer F344 rats with myocardial infarction (MI) attenuated several aspects of cardiac dysfunction and adverse remodeling in the post-MI setting, as revealed by the improvement in passive left ventricular compliance, increased endothelial cell density, reduced myocyte hypertrophy, and collagen 1 expression [267]. Since endothelial integrity and restitution of the lost cardiac microvasculature observed in MI are essentially mediated by stromal-derived factor (SDF1*α*), a chemokine secreted by ischemic tissue but rapidly degraded by DDP-4, it is possible that the benefit following MI in the diabetic animals is beyond its effect on glycemia. However, since DPP-4 activity determines the systemic and local concentrations of SDF-1*α* and the mobilization to the injured sites of stem cells involved in endothelial repair and angiogenesis, further studies are needed to clarify whether DPP-4 inhibition is able to reverse bone marrow dysfunction induced by diabetes and improve microvascular health in the ischemic tissue [109].

Regarding clinical data, McCormick et al. (2014) have recently shown that chronic DPP-4 inhibition with sitagliptin 100 mg/day for 4 weeks protected against ischemic left ventricular dysfunction during dobutamine stress in patients with T2DM (19 patients) and coronary artery disease, possibly by GLP-1-mediated cardioprotection on ischemic regional wall segments [270]. However, randomized studies involving large patient cohorts are required to ascertain whether these effects translate into an improvement in clinical outcomes.

#### 4.3.2. Vildagliptin

Liu et al. have shown that vildagliptin treatment for 24 weeks led to an improvement in renal lesions, as revealed by inhibition of interstitial expansion, glomerulosclerosis, and thickening of the glomerular basement membrane in T1DM rats [271]. Vildagliptin significantly reduced renal DPP-4 activity and increased plasma active GLP-1 levels, which probably prevented oxidative DNA damage mediated by suppression of TGF-*β*1 and renal cell apoptosis by activating GLP-1R and modulating the second messenger cAMP [271]. In a T2DM animal model, the ZDF rat, vildagliptin treatment did not affect glucose levels or proteinuria, but it significantly decreased glomerulosclerosis and restores myogenic constriction of intrarenal arteries, suggesting that this DPP-4 inhibitor protects diabetic rats from loss of renal vascular reactivity and attenuates renal sclerosis independent of effects on blood glucose or proteinuria in T2DM [272].

Few data exist concerning the effects of the DPP-4 inhibitor vildagliptin on the kidney of diabetic patients. Tani et al. (2013) have assessed the effect of vildagliptin (50 mg bid for 8 weeks) on atherogenic low-density lipoprotein (LDL) heterogeneity and albuminuria in diabetics [273]. Treatment of T2DM with vildagliptin for 8 weeks decreased significantly the serum small dense LDL levels by about 9% and the urinary albumin-to-creatinine ratio (UACR) by about 45%, suggesting that vildagliptin might prevent cardiovascular disease by improving LDL heterogeneity and improve renal function by decreasing albuminuria.

Accordingly, vildagliptin (3 mg/kg/day) treatment for 12 weeks suppressed the expression of TGF-*β* in the aorta of diabetic rats, by attenuating the deleterious effects of advanced glycation end products (AGEs) and their receptor RAGE axis, with suppression of oxidative stress generation and inflammation in aorta of diabetic and obese Otsuka Long Tokushima Fatty (OLEFT) rats [274]. Wang et al. investigated the impact of DPP-4 inhibition on cardiac microvascular injury in diabetes and the underlying mechanism involved. STZ-induced diabetic rats treated with vildagliptin (1 mg/kg/day) for 12 weeks improved cardiac function and glucose uptake, suggesting that GLP-1 could protect the cardiac microvessels against oxidative stress, apoptosis, and the resultant microvascular barrier dysfunction in diabetes, en route to improved cardiac diastolic function and cardiac glucose metabolism. The protective effects of GLP-1 were dependent on downstream inhibition of Rho through a cAMP/PKA-dependent manner, which may result in the cardioprotective effect on cardiovascular remodelling associated with oxidative stress [275].

There is only one experimental study examining the effect of vildagliptin on retinal injury in diabetes. OLEFT rats at 22 weeks of age treated with vildagliptin (3 mg/kg) for another 10 weeks presented a significant inhibition of the increase in body weight and decreased average fasting blood glucose. Vildagliptin also inhibited inflammatory and thrombogenic reactions in the retinas of obese T2DM rats, suggesting that it may play a protective role against diabetic retinopathy [276].

#### 4.3.3. Saxagliptin and Other DPP-4 Inhibitors

Tahara et al. (2009) performed a comparative study investigating the antidiabetic potency and duration of several DPP-4 inhibitors (0.1–3 mg/kg) in rats with mild diabetes (streptozotocin-nicotinamide-induced models) [277]. The potency order and duration of action for plasma DPP-4 inhibition and glucose tolerance improvement were as follows: saxagliptin > vildagliptin = sitagliptin. In this report, vildagliptin and sitagliptin improved glucose tolerance through increased insulin and GLP-1 levels in plasma 8 h at a dose of 1 mg/kg. Furthermore, saxagliptin potently improved glucose tolerance at a dose of 0.3 mg/kg, reflecting the long half-life of the enzyme complex formed by saxagliptin [277]. These data suggest that the effects are mediated through glucose-dependent insulinotropic action via an increase in the GLP-1 level. Kodera et al. (2014) recently reported renoprotective effects of a DPP-4 inhibitor compound (PKF275-055) in early stages of diabetic nephropathy in rats due to anti-inflammatory actions [278].

Regarding clinical data, the large, randomized, placebo-controlled SAVOR-TIMI 53 (*Saxagliptin Assessment of Vascular Outcomes Recorded in Patients with Diabetes Mellitus*) trial showed that T2DM patients with cardiovascular complications under saxagliptin treatment were significantly more likely, when compared to placebo-treated patients, to have improved albumin-to-creatinine ratio (10.7% in the saxagliptin group and 8.7% in the placebo group) and less likely to have worsening ratio (13.3% in the saxagliptin group and 15.9% in the placebo group), suggesting a protection on albumin excretion rate [225]. Whether the effects observed are attributed, at least partially, to a better glucose control, or to a direct effect of saxagliptin, as suggested by preclinical data, remains to be fully clarified.

A recent double-blind study using 50 patients with T2DM (mean duration of 4 years) without signs of retinopathy has shown that saxagliptin administration (5 mg for 6 weeks) significantly reduced retinal capillary blood flow, suggesting that it is able to reverse early hemodynamic and vascular remodeling processes in T2DM [279].

Concerning other DPP-4 inhibitors, Aroor et al. (2013) have demonstrated that linagliptin treatment on ZDF rats for 8 weeks is beneficial in blunting obesity-associated cardiac diastolic dysfunction in the prediabetic state [280]. Furthermore, using the same approach, Nistala et al. (2014) have reported that DPP-4 inhibition with linagliptin improved proteinuria along with filtration barrier remodelling, circulating, and kidney tissue DPP-4 activity, increased active GLP-1 as well as SDF-1*α*, and improved oxidant markers and the podocyte-specific protein nephrin, suggesting that targeting DPP-4 may have a beneficial effect on the initial stages of obesity-related kidney disease [281].

Regarding human data, Groop et al. (2013) analyzed data from 4 similarly designed (randomized, double-blind, and placebo-controlled) phase III trials, involving 217 individuals with T2DM and prevalent albuminuria under treatment with renin-angiotensin-aldosterone system inhibitors [282]. The authors showed that at 24 weeks linagliptin (5 mg/day) was able to reduce (32%) urinary albumin-to-creatinine ratio (UACR) when compared with patients (6%) randomized to receive placebo. The lack of correlation between the degree of UACR reduction and the level of change in HbA1c and SBP suggests that the improvement in urinary albumin excretion by linagliptin could be independent of glycemic and BP controls. The MARLINA-T2DM (efficacy, safety, and modification of albuminuria in type 2 diabetes subjects with renal disease with linagliptin) trial is ongoing, in order to evaluate the albumin-lowering potential of linagliptin in T2DM patients with renal impairment [283].

Sakata et al. (2013) reported an improvement in AGE-RAGE (advanced glycation end products-advanced glycation end products) axis and a reduction in albuminuria in Japanese T2DM patients treated with alogliptin (25 mg once daily) during 12 weeks [284]. Recently, Fujita et al. (2014) performed a small, nonrandomized, crossover study with sitagliptin and alogliptin administration on top of angiotensin receptor blockers treatment in T2DM patients with early nephropathy [285]. Four weeks of alogliptin (25 mg/day) treatment after 4 weeks of sitagliptin (50 mg/day) therapy was able to significantly reduce urinary albumin levels, whereas HbA1c, blood pressure, serum lipids, and estimated glomerular filtration rate were found to be unchanged. The authors also observed a reduction in the urinary oxidative marker 8-hydroxy-20-deoxyguanosine and an increase in urinary cAMP level and serum SDF-1a level, suggesting a benefit of alogliptin treatment against early diabetic nephropathy related to antioxidative stress pathways.

To conclude, clinical data addressing macro- and microvascular endpoints in T2DM patients are warranted to provide information whether the promising preclinical findings can be translated into clinical benefit. Ongoing clinical trial will probably shed light not only on the extrapancreatic benefits, but also on safety of DPP-4 inhibitors.

**Figure 2:**
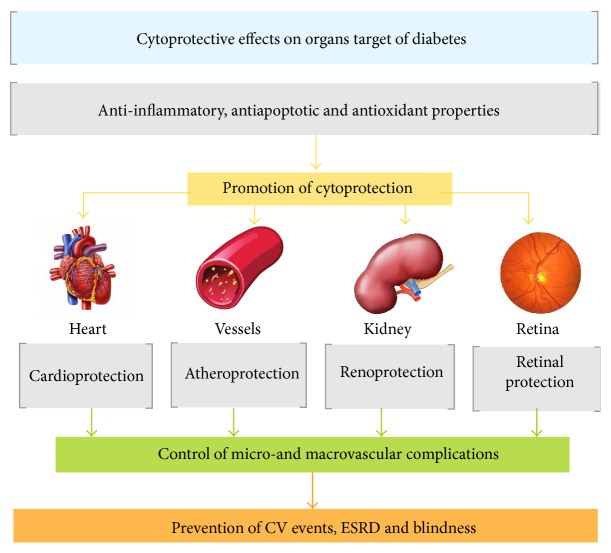
Putative cytoprotective effects of dipeptidyl peptidase-4 inhibitors on organs/tissues targeted by diabetes, including the heart, vessels, kidney, and retina, that are associated with serious diabetic complications.

Figure 2 schematically represents the cytoprotective effects of DPP-4 inhibitors on extrapancreatic organs/tissues targeted by diabetes, including the heart, vessels, kidney, and retina, which could be important to control the severe micro- and macrovascular complications found in diabetic patients, including cardiovascular events, end-stage renal disease (ESRD), and blindness. Our experimental data is suggestive of those putative protective effects and is in line with other previous studies already discussed above. If further confirmed in a near future, namely, in human organs/tissues, DPP-4 inhibitors might represent a key step forward in the management of T2DM and its serious complications.

## 5. Concluding Remarks

T2DM treatment based on the “incretin defect” is a physiological method. DPP-4 inhibitors (gliptins) have unique benefits that complement and extend the current available therapeutic options for T2DM. Incretin-based therapies can modify various steps in the pathophysiology of T2DM, including hypersecretion of glucagon, gastric emptying, postprandial hyperglycaemia, and possibly chronic dysfunction of pancreatic *β* cells. Overall, gliptins are efficient at reducing plasma glucose, similar to other therapeutic groups, and its use can be made in a combined form with other antidiabetic agents with distinct mechanism of action, in particular with metformin, the most widely used combination. The use of DPP-4 inhibitors has therapeutic benefits, such as improving the secretion of insulin and glucose-dependent suppression of glucagon synthesis. Other benefits, including reduction of blood pressure and amelioration of lipid profile, have also been described.

Furthermore, DPP-4 inhibitors have the ability to improve metabolic control in T2DM, with minimal risk of adverse effects, including hypoglycaemia, which is very important for the treatment of a large group of diabetic patients, including the elderly ones. The putative association of DPP-4 therapy with development of pancreatitis and pancreatic cancer remains to be confirmed. Although several lines of evidences do not support such association, current evidence is not definitive and we should undoubtedly remain vigilant. In any case, currently the balance of evidence is strongly in support of benefits far outweighing potential risks.

One of the most relevant and innovative aspects of these new therapies is that they seem to be able to protect the pancreas from progression of deterioration that inevitably seems to occur with the current treatments available, especially due to the ability of DPP-4 inhibitors to protect or even regenerate the pancreatic *β* cell by mechanisms related to their antiapoptotic and proproliferative properties. This possibility raises the question when DPP-4 therapy should be started. T2DM is characterized by a progressive loss of *β* cells mass and function that is associated with insulin resistance, which starts early in the prediabetic states. These defects are followed by a significant decrease in the incretin effect, possibly due to abnormalities in secretion and action of GLP-1. In this sense, DPP-4 inhibitors will theoretically be useful in early stages of diabetes, when the patient still retains a *β* cell population capable of responding to GLP-1 stimulation. The possibility has been tested, but the current knowledge is scarce to fully recommend such use. On the other hand, given the complementary effects of DPP-4 and insulin, this association has been tested and in the near future additional data obtained from larger studies should better clarify the benefits and risks of this association in some subpopulations of diabetic patients.

One of the most interesting and innovative aspects of incretin-based therapies, including DPP-4 inhibitors, is the putative cytoprotective effect on extrapancreatic organ or tissues target by diabetes, such as the heart, vessels, kidney, and retina. Our group has shown beneficial effects of sitagliptin not only on the pancreas but also on the heart, kidney, and retina in animal models of T1DM and T2DM [116, 252, 255, 256, 258], which are in line with other studies focused on cardiomyopathy, nephropathy, and retinopathy. If these potential extrapancreatic effects observed in experimental studies can be confirmed and reinforced in humans, then DPP-4 inhibitors could become a preferred treatment for T2DM due to their ability to modify the natural history of disease by preventing its evolution to more serious stages, as well as due to the protection afforded against evolution of diabetes organ-target complications, thus preventing cardiovascular events, ESRD, and progressive loss of vision. It remains to be seen, however, whether these benefits, mainly obtained from preclinical studies, will translate into clinical outcomes (such as reduction in cardiovascular events and mortality, as well as amelioration of nephropathy and retinopathy) in large-scale studies.

However, to conclude, randomized studies involving large patient cohorts are required to ascertain whether these effects translate into an improvement in clinical outcomes.

## Figures and Tables

**Table 1:** Main features of non-incretin-based antidiabetic drugs for T2DM treatment.

Class	Mechanisms of action	Effects/advantages	Adverse reactions	ΔHbA1c (−%)^∗^
Biguanides	Decrease hepatic glucose production and gluconeogenesis Decrease intestinal glucose absorption Increase peripheral glucose utilization	Reduce blood glucose levels in hyperglycaemic state only Reduce lipid levels Cause modest reduction of body weight Perform oral administration	Nausea and vomiting Diarrhoea Lactic acidosis	1.0–2.0

Sulfonylureas	Increase pancreatic insulin secretion Decrease or unchanged plasma insulin levels	Reduce blood glucose levels in hyperglycaemic and normoglycemic states Increase plasma insulin levels Perform oral administration	Increased body weight Hypoglycaemia Nausea and vomiting	1.0–2.0

Thiazolidinediones	Decrease hepatic glucose production and gluconeogenesis Increase peripheral glucose utilization	Reduce blood glucose levels in hyperglycaemic state only Reduce lipid levels (triglycerides) Have possible benefits on cardiovascular risk factors Perform oral administration	Increased body weight Anaemia Oedema Congestive heart failure in susceptible individuals	0.5–1.4

Meglitinides	Increase pancreatic insulin secretion	Reduce blood glucose levels in hyperglycaemic and normoglycemic states Reduce postprandial glucose excursions Have no significant effects on lipid levels Perform oral administration	Hypoglycaemia Increased body weight Diarrhoea	0.5–1.5

*α*-glucosidase inhibitors	Perform reversible inhibition of *α*-glucosidase enzymes Decrease digestion of complex carbohydrates Delay glucose absorption	Reduce blood glucose levels in hyperglycaemic state only Have no significant effects on lipid levels Have no effects on body weight Perform oral administration	Abdominal pain Elevation of liver enzymes Diarrhoea	0.5–0.8

Sodium-glucose cotransporter 2 inhibitors	Perform inhibition of renal reabsorption of glucose, thus increasing urinary glucose excretion	Reduce plasma glucose Have beneficial effects on body weight and blood pressure Have low risk of hypoglycaemia	Risk of urinary and genital tract infections Requirement of regular monitoring of renal function and kalemia	0.5–0.8

Insulin	Replace endogenous insulin	Reduce blood glucose levels in hyperglycaemic and normoglycemic states Perform subcutaneous administration	Hypoglycaemia Increased body weight	1.5–3.5

^∗^HbA1c variations (negative %) are mean values.

**Table 2:** Main features of DPP-4 inhibitors versus other (non-incretin-based) oral antidiabetic drugs, Table 1.

Class	Mechanisms of action	Effects/advantages	Adverse reactions	ΔHbA1c (%)
DPP-4 inhibitors	Inhibit metabolism by DPP-4 enzyme Increase the endogenous bioactive form of GLP-1	Reduce blood glucose levels and the postprandial glucose excursion Have no effects on body weight Have possible effects on pancreatic *β* cells mass Perform oral administration	Abdominal pain Nausea and vomiting Diarrhoea Nasopharyngitis Respiratory infections Headache	0.5–1.0

**Table 3:** Main characteristics of the DPP-4 inhibitors already approved for use in the US and/or EU market: sitagliptin, vildagliptin, saxagliptin, alogliptin, and linagliptin.

	Sitagliptin	Vildagliptin	Saxagliptin	Alogliptin	Linagliptin
Dosing	100 mg qd	50 mg bid	5 mg qd	25 mg qd	5 mg qd

Max. DPP-4 inhibition (%)	±97	±95	70–80	>90	>90

Selectivity for DPP-4	High	High	Moderate	High	High

^∗^HbA1c reduction (%)	0.5–1.0	0.9 (mean value)	0.5–1.0	0.6 (mean value)	0.5–0.7

Hypoglycaemic risk	Low	Low	Low	Low	Low

Half-life compound (*t*_1/2_, h)	±12	1.5–3	±2.5	11–22	>100

Bioavailability (%)	±87	±85	±67	—	±30

Metabolism/ elimination	Renal excretion almost unchanged (70–80% parent)	Renal excretion (±26% parent and ±55% as metabolite obtained after hydrolysis)	Liver metabolized to active metabolite by P450 3A4/5 and renal excretion (12–29% unchanged parent and 21–52% as metabolite)	Renal excretion almost unchanged parent (60–70%)	Biliary excretion almost unchanged (>70% unchanged parent) and renal (<6%)

Adapted from [93–99] using available information/knowledge. ^∗^HbA1c variations vary between studies and depend on baseline levels. Values presented are the range or mean value calculated from the studies available.
